# Investigation of the In-Vivo Cytotoxicity and the In Silico-Prediction of MDM2-p53 Inhibitor Potential of *Euphorbia peplus* Methanolic Extract in Rats

**DOI:** 10.3390/toxins11110642

**Published:** 2019-11-04

**Authors:** Yasmina M. Abd-Elhakim, Mohamed Abdo Nassan, Gamal A. Salem, Abdelkarim Sasi, Adil Aldhahrani, Khaled Ben Issa, Amany Abdel-Rahman Mohamed

**Affiliations:** 1 Department of Forensic Medicine and Toxicology, Faculty of Veterinary Medicine, Zagazig University, Zagazig 44511, Egypt; 2 Laboratories Technology Department, Turabah University College, Taif University, Turabah 21995, Saudi Arabia; moh_nassan@yahoo.com; 3 Department of Pathology, Faculty of Veterinary Medicine, Zagazig University, Zagazig 44511, Egypt; 4 Department of Pharmacology, Faculty of Veterinary Medicine, Zagazig University, Zagazig 44511, Egypt or; 5 Department of Pharmacology, Faculty of Pharmacy, Misurata University, Misurata 2478, Libya; 6 Department of Chemistry, Faculty of Pharmacy, Misurata University, Misurata 2478, Libya; Kareem.sasi@gmail.com; 7 Laboratories Technologie Department, Turaba University College, Taif University, Turabah 21995, Saudi Arabia; adildh@hotmail.com; 8 Pharmaceutical and Industrial Department, Faculty of Pharmacy, Misurata University, Misurata 2478, Libya; khaledbenissa87@gmail.com

**Keywords:** *Euphorbia peplus*, MDM2-p53, apoptosis, anticancer, di-(2-ethylhexyl) phthalate

## Abstract

This study explored the probable in vivo cardiac and renal toxicities together with in silico approaches for predicting the apoptogenic potential of *Euphorbia peplus* methanolic extract (EPME) in rats. Cardiac and renal injury biomarkers were estimated with histopathological and immunohistochemical evaluations of both kidney and heart. The probable underlying mechanism of *E. peplus* compounds to potentiate p53 activity is examined using Molecular Operating Environment (MOE) docking software and validated experimentally by immunohistochemical localization of p53 protein in the kidney and heart tissues. The gas chromatography/mass spectrometry analysis of *E. peplus* revealed the presence of nine different compounds dominated by di-(2-ethylhexyl) phthalate (DEHP). Significant elevations of troponin, creatine phosphokinase, creatine kinase–myocardium bound, lactate dehydrogenase, aspartate transaminase, alkaline phosphatase, urea, creatinine, and uric acid were evident in the EPME treated rats. The EPME treated rats showed strong renal and cardiac p53 expression and moderate cardiac TNF-α expression. Further, our in silico results predicted the higher affinity and good inhibition of DEHP, glyceryl linolenate, and lucenin 2 to the MDM2-p53 interface compared to the standard reference 15 a compound. Conclusively, EPME long-term exposure could adversely affect the cardiac and renal tissues probably due to their inflammatory and apoptotic activity. Moreover, the in silico study hypothesizes that EPME inhibits MDM2-mediated degradation of p53 suggesting possible anticancer potentials which confirmed experimental by strong p53 expression in renal and cardiac tissues.

## 1. Introduction

For various communities and civilizations throughout the world, herbal medicines were known as a preferable solution for many health disorders and to avoid problems concerning reluctance toward and the negative impacts of the current antimicrobial medications [1]. The World Health Organization established that 80% of the population of developing countries, about 3.3 billion people, use medicinal plants regularly without prescription [2]. Depending on their use for long durations, many people could suppose that plants used in customary medicine are of low toxicity since they belong to natural sources [3]. Nevertheless, the most recent surveys have revealed that many medicinal plants applied in folk remedies showed adverse effects [4,5]. Hence, customary herbal medicines are getting substantial consideration in health debates globally [6].

*Euphorbia* is a large genus of the *Euphorbiaceae* family that comprises more than 2000 species growing nearly in most climatic conditions all over the world [7]. Many favorable pharmacological properties have been identified for some *Euphorbia* species like antioxidant, anti-inflammatory, anticancer, and hepatoprotective effects [8,9,10]. Nonetheless, some hazardous impacts have been recognized for other species. For instance, Adedapo et al. [11] has shown that *E. hirta* has potentially deleterious effects on the serum biochemistry of rats and indicated that the existence of this plant in the pasture could represent a toxicity source to livestock. Also, Al-Sultan and Yehia [12] have documented the damaging effect of *Euphorbia heliscopia* in different rats’ organs.

*Euphorbia peplus* is initially native to Europe and North Africa and currently arises worldwide because of its fast spread [13]. For centuries, *E. peplus* has been adopted as a topical medication for the curing of many skin disorders in customary medicine schemes worldwide [14,15]. The analgesic, antimicrobial, antipyretic, and anti-inflammatory effects of *E. peplus* have been previously reported [16,17]. Recently, some non-polar secondary metabolites isolated from *E. peplus* plant have been used for the treatment of leishmaniasis [18]. The LD_50_ of most euphorbia species was estimated to be more than 5000 mg/kg [3,19]. Additionally, the earlier studies reported that the doses up to 2000 mg/kg were devoid of toxic effects in acute toxicity studies of other euphorbia species [20]. However, recently, Moustafa et al. [21] reported that the oral dosing of 500mg/kg b. wt *E. peplus* methanolic extract twice weekly for 65 days in rats induced a significant decline in weight of seminal vesicle and testis, sperm cell concentration, sperm motility, and antioxidant enzymes, testosterone, and LH hormones levels. 

The heart is a very vital organ in the body and cardiac dysfunction means serious concerns for the whole organism. Since only a fine line of discrepancy presents itself between a medicinal plant being cardioprotective and it is being toxic at times, accurate monitoring is highly needed. Hence, various indicators for the assessment of the cardioprotective or cardiotoxic effects of medicinal plants have been adopted. Modulation of enzyme biomarkers, as well as histopathological evaluations, often are employed in these assessments [22]. Kidney function investigation is very essential in the toxicity assessment of plant extracts as it is essential for the being of an organism [23]. Earlier reports have documented the renal injury following the ingestion of some Euphorbia species including *Euphorbia paralias* [24], *Euphorbia. heliscopia* [12], and *Euphorbia pekinensis* [25]. However, little is known about the probable cardiotoxic and nephrotoxic effects of the exposure to *E. peplus*.

Apoptosis has been proposed as an underlying mechanism of the cytotoxic effects of several *Euphorbia* species including *Euphorbia hirta* [26], *Euphorbia lunulata* [27], and *Euphorbia fischeriana* [28]. However, little is known on the probable role of apoptosis in *E. peplus* induced cytotoxicity. The tumor suppressor protein p53 has a key role in cell apoptosis, autophagy, cell division, and growth [29,30,31,32]. The hindrance of p53 activity can lead to the evolution of cancer and tumor development [33]. In normal unstressed cells, p53 is negatively controlled by physical collaboration with murine double minute 2 (MDM2) protein [34]. This interaction renders the p53 unstable protein with low cellular level owing to continuous E3 ubiquitin ligase activity of MDM2 that promotes p53 ubiquitination and further proteasome degradation [35]. The feedback regulatory mechanism between p53 and MDM2 protein exerts an important role in suppressing the p53 activity under physiological and stressful conditions [36]. It has been observed that overexpression of MDM2 could deactivate the p53 oncogenic suppressor function and induce the failure of apoptosis and proliferation of cancer cells [37]. It is also well proven that disrupting p53/MDM2 protein binding would restore wild-type p53 protein activity and subsequently enhance the apoptotic pathway in cancer and even normal cells [38], which may be valuable in treating different types of cancer cell pathway proliferation.

In molecular docking studies, Molecular Operating Environment (MOE) has been considered a highly accurate predictive tool of the biological activity of molecules that enable the rational design of pharmaceutical agents [39,40]. In particular, using MOE software led to the discovery of novel Mdm2-p53 interaction inhibitors that could work as key tool compounds for cellular and animal studies [41,42].

Based on the wide use of *E. peplus* in traditional practices and the scarcity of the studies evaluating its probable toxic impacts, the current study was performed to assess the impacts of subchronic exposure to the methanolic extract *of E. peplus* on cardiac and renal injury indices and the histopathological changes of both cardiac and renal tissues. Moreover, the marker of inflammation and apoptosis were immunohistochemically evaluated. Additionally, the chemical characterization of the main constituents of the extract was completed, and further in silico approaches were simulated for predicting the potential of these compounds as MDM2 inhibitors to reveal a new approach to the mechanisms of induction of apoptosis in normal and cancer cells. 

## 2. Results

### 2.1. Experimental Observations

During the experimental period, no deaths or abnormal signs were observed in the control or treated groups.

### 2.2. GC-MS Profile of EPME

**Figure 1:**
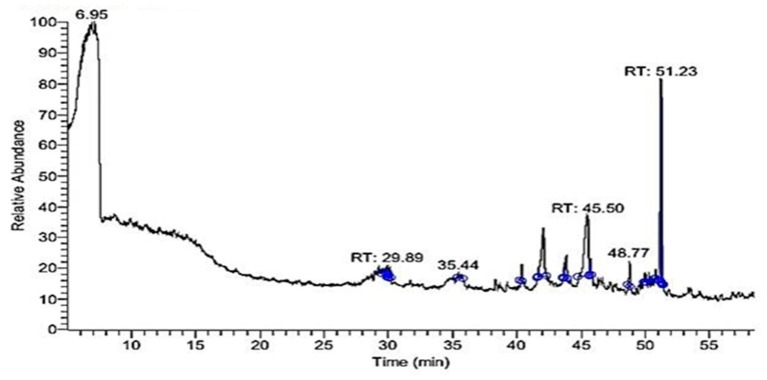
GC-MS chromatogram of *Euphorbia peplus* methanolic extract.

Table 1 and Figure 1 showed the chief constituents of *E. peplus* methanolic extract (EPME), their relative percentage of the total peak area and retention times following GC-MS analysis. A total of 19 signals have been detected. The 6.95 peak is corresponding to the solvent used. However, we integrated the analysis after the time of appearance of this peak because of its high relative intensity which could minimize the peak area % of other components. The data indicate that the major components of EPME were Di-(2-ethylhexyl) phthalate (36.07%), oleic acid (27.82%), hexadecanoic acid (15.04%), lucenin 2 (4.54%), and à-Gurjunene (4.36%).

### 2.3. Effects of EPME on Cardiac Injury Markers

The effects of oral administration of EPME for 65 days on rats’ serum cardiac injury markers are shown in Table 2. A pronounced elevation in serum levels of troponin, CPK, and CKMB were observed in the EPME-treated group (about six-fold, one-fold, and two-fold, respectively) compared to the control group. Moreover, a significant increase in serum levels of LDH, AST, and ALP was observed in the EPME-treated rats (126.9%, 18.8%, and 93.6%, respectively) relative to the control rats.

### 2.4. Effects on EPME on Markers of Kidney Injury 

**Figure 2:**
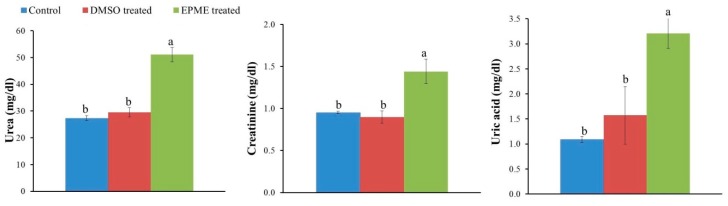
Effects of *Euphorbia peplus* methanolic extract (500 mg/kg b. wt. orally two times per week for 65 days) on kidney injury biomarkers (urea, creatinine, and uric acid) in rats. The values shown are means ± SE (n = 5). Bars with different letters (**a**,**b**) significantly differ from one another. (**a**) denotes the highest mean. The *P*-values were 0.001, 0.005, and 0.008 for creatinine, urea, and uric acid, respectively.

As shown in Figure 2, the rat exposed to EPME for 65 days displayed a significant elevation in the levels of urea (*P* = 0.001), creatinine (*P* = 0.005), and uric acid (*P* = 0.008) by 86.8%, 51.6%, and 194.3%, respectively compared to the control group.

### 2.5. Histopathological and Immunohistochemical Findings

**Figure 3:**
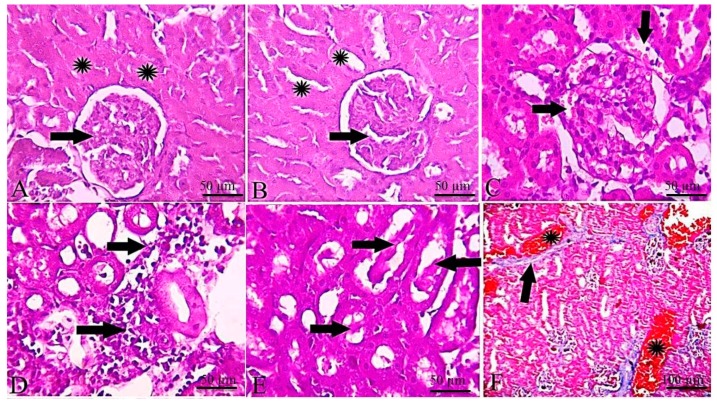
Representative photomicrographs of H&E-stained kidney sections. (**A**) control group showing normal tissue architecture represented by normal glomeruli (arrow) and renal tubules (*). (**B**) DMSO administered group not showing any tissue changes with normal glomerular (arrow) and tubular structure (*). (**C**) EPME treated group showing congestion of glomerular and periglomerular capillaries (arrows). (**D**) Peritubular round cell infiltration (arrows). (**E**) Hyaline casts inside the lumina of renal tubules (arrows). (**F**) Congestion of renal blood vessels (*) with moderate perivascular fibrosis (arrow) (Masson trichrome stain).

**Figure 4:**
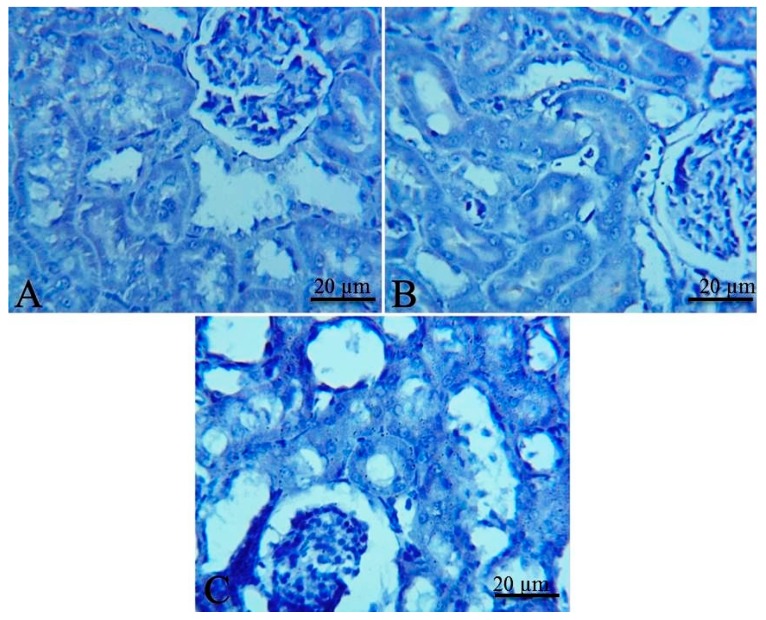
Representative photomicrographs of immunohistochemical staining for Bax in rat kidneys. (**A**–**C**) Control, DMSO, and EPME treated group showing the absence of expression of Bax in the renal tissue.

The kidney of the control group showed normal tissue architecture represented by normal glomeruli and renal tubules (Figure 3A). The kidney of the DMSO administered group didn’t show any tissue changes with normal glomerular and tubular structure (Figure 3B). The kidney of EPME treated group showed congestion of glomerular and periglomerular capillaries (Figure 3C). Peritubular round cell infiltration was detected in the interstitium (Figure 3D). Hyaline casts were observed inside the lumina of renal tubules (Figure 3E). Congestion of renal blood vessels was detected with moderate perivascular fibrosis (Figure 3F). The kidney of control, DMSO, and EPME treated group showed the absence of expression of Bax in the renal tissue (Figure 4).

**Figure 5:**
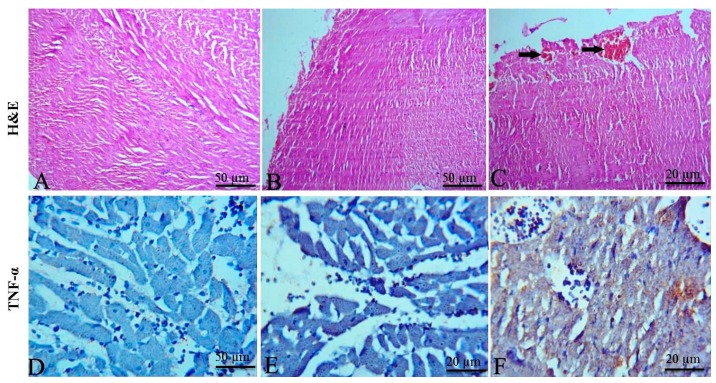
Representative photomicrographs of H&E-stained heart sections and immunohistochemical staining for TNF-α in rat cardiac tissues. (**A**) Control group showing normal tissue architecture. (**B**) DMSO administered group not showing any tissue changes with normal myocytes. (**C**) Heart of EPME treated group showing mild congestion of coronary vessels (arrows). (**D**) Control group showing the absence of expression of TNF-α. (**E**) DMSO administered group didn’t show any expression of TNF-α in the cardiac myocytes. (**F**) Heart of EPME treated group showing moderate expression of TNF-α in the cardiac myocytes.

No significant changes were detected in the heart of control and treated groups except for mild congestion of coronary vessels and cardiomyocytes necrosis in EPME treated group (Figure 5A–C). The heart of the control (Figure 5D) and DMSO administered group (Figure 5E) didn’t show any expression of TNF-α, while the heart of the EPME treated group showed moderate expression of TNF-α in the cardiac myocyte (Figure 5F). 

**Figure 6:**
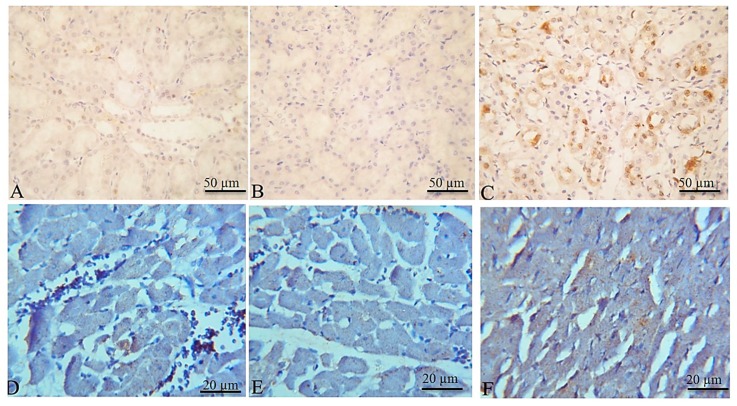
Representative photomicrographs of immunohistochemical staining for p53 in rat kidneys (**A**–**C**) and hearts (**D**–**F**). (**A**) Kidney of control group showing rare expression of p53 in renal tubular tissue. (**B**) Kidney of DEMSO administered group with a mild expression of p53 in renal tubular tissue. (**C**) Kidney of EPME treated group showing increased expression of p53 in renal tissue. (**D**) Heart of control group showing a mild expression of p53 in cardiac myocytes. (**E**) Heart of DEMSO administered group with a mild expression of p53 in cardiac tissue. (**F**) Heart of EPME treated group showing increased expression of p53 in cardiac myocytes.

The kidney of the control and DEMSO administered group showed mild expression of p53 in renal tubular tissue (Figure 6A,B respectively). On the other hand, the kidneys of the EPME treated group showed increased expression of p53 in renal tissue (Figure 6C). The heart of the control and DEMSO administered group showed mild expression of p53 in cardiac myocytes (Figure 6D,E respectively). Meanwhile, the cardiac tissue of the EPME treated group showed increased expression of p53 in cardiac myocytes (Figure 6F). 

### 2.6. Molecular Docking Results

**Figure 7:**
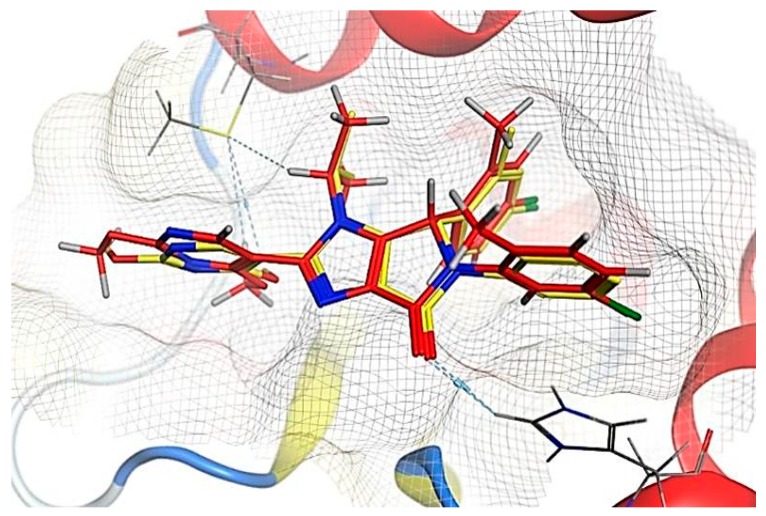
The RMSD between the original poses (yellow) and the redocked poses (red). RMSD value (0.49 Å) between the redocked pose (red) and the original pose (yellow) of the native ligand (compound15a) in MDM2 active site (PDB: 6GGN).

The root mean square deviation (RMSD) value for the redocked native ligand (Figure 7) was 0.49 Å. The redocked compound 15a was superimposed and this inhibitor showed that it perfectly aligns with the original position in the binding cavity of the MDM2.

The docking results between MDM2 with the nine compounds identified in *Euphorbia* revealed that seven of the compounds (Di-(2-ethylhexyl) phthalate, oleic acid, vitamin A aldehyde, 9,12-octadecadienoic acid (Z,Z)-, methyl ester (methyl linoleate), glyceryl linolenate, lucenin 2, and oxiraneundecanoic acid, 3-pentyl-, methyl ester with binding energies of −12, −12, −9, −10, −14, −20, −12 kcal/mol, respectively) showed higher binding affinities to MDM2 compared with a known inhibitor (15a; −8.172 kcal/mol; Table 3). However, the binding energies of palmitic acid and α-gurjunene “humulene” to MDM2 were lower when compared with the MDM2 inhibitor ligand, 15a compound.

**Figure 8:**
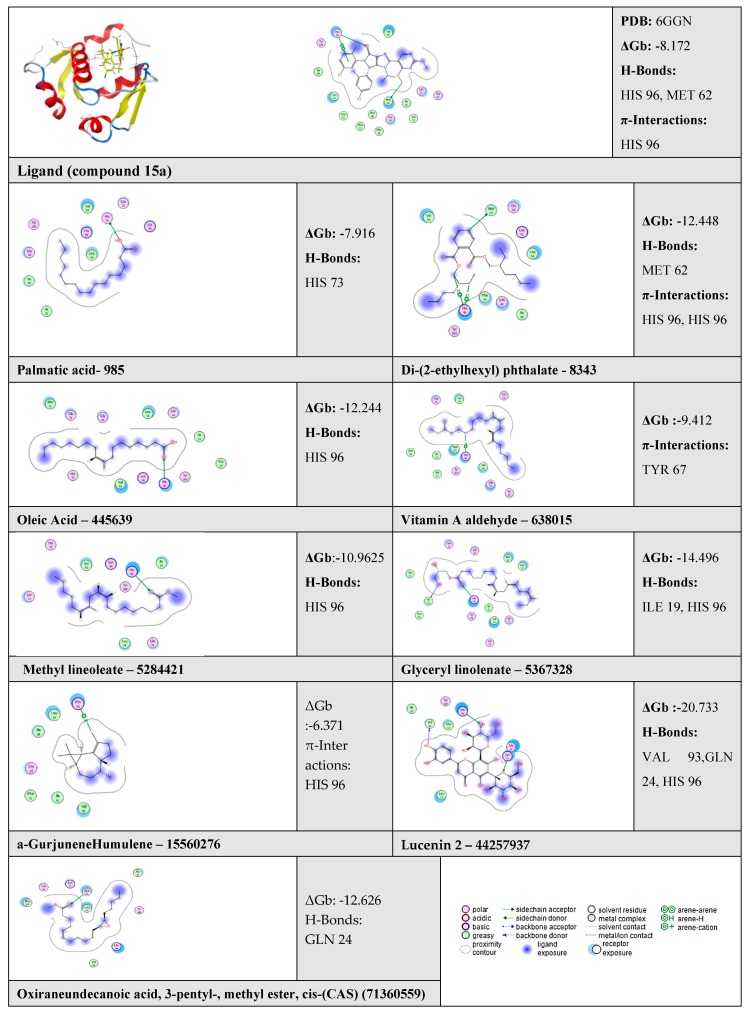
The docked poses of *Euphorbia* compounds and 15 a compound with the p53 binding site of the MDM2 protein.

**Figure 9:**
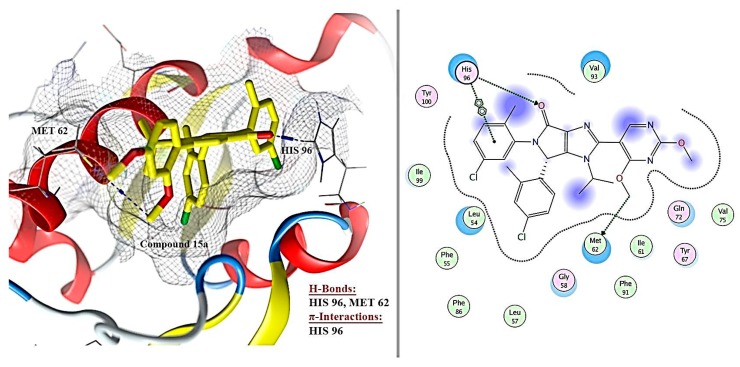
Shows the binding mode (interactions) of compound 15a with the p53 binding site of the MDM2 protein (Pdb: 6GGN).

Visual examination of the docked position of 15a ligand showed three interactions with MDM2 protein (Figure 7, Figure 8 and Figure 9). It forms hydrogen bond interaction with HIS 96 and MET 62 and also produces π-π interactions with HIS 96.

**Figure 10:**
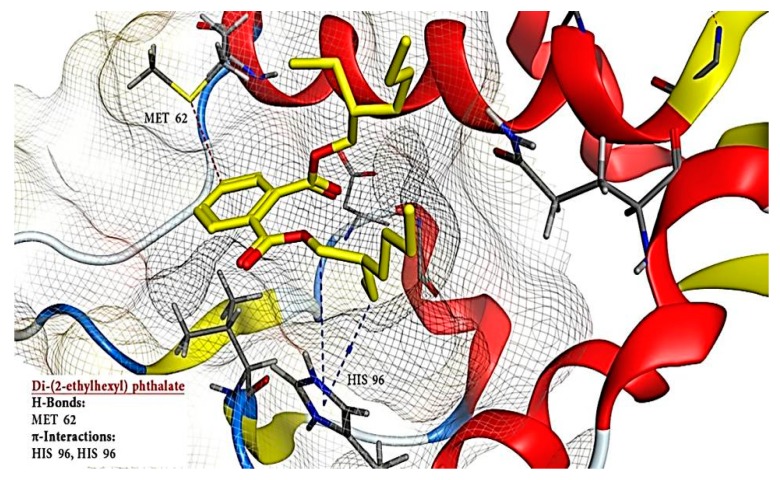
Three-dimensional (3D) structure of MDM2 in complex with Di-(2-ethylhexyl) phthalate. The H-bond is indicated with a dashed line.

**Figure 11:**
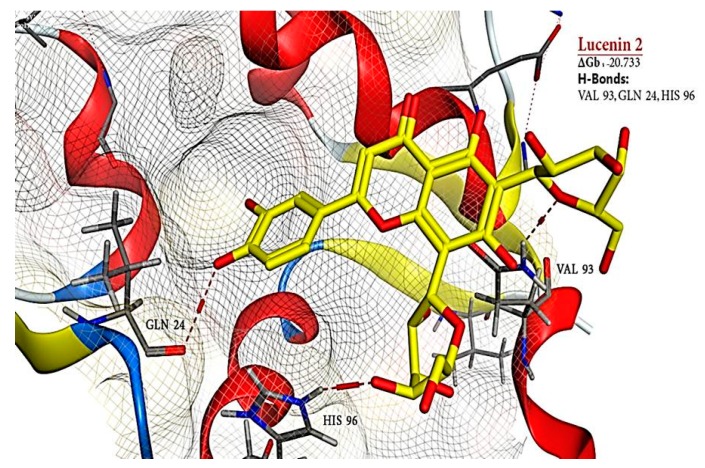
Three-dimensional (3D) structure of MDM2 in complex with lucenin 2. The H-bond is indicated with a dashed line.

Docked compounds showed different binding modes in the active site including hydrogen bonds, in addition to H-π and π-π interactions. These compounds bind to the MDM2 protein by interacting with key amino acid residues, such as HIS 73, MET 62, HIS 96, TYR 67, ILE 19, VAL 93, and GLN 24. (Table 3 and Figure 8). The interactions of lucenin 2 and Di-(2-ethylhexyl) phthalate inside MDM2- p53 binding pocket are presented in Figure 10 and Figure 11. 

## 3. Discussion

In this research, the chemical composition of EPME has been recognized using the GC-MS technique to report the incidence of nine compounds, predominated by Di-(2-ethylhexyl) phthalate (DEHP). DEHP has been identified as the main component in other plants like *Nigella glandulifera Freyn* [43], *Calotropis gigantea* [44], *Mallotus tetracoccus* [45], and *Launaea resedifolia* [46]. DEHP elicited several health disorders like endocrine disturbances [47], reprotoxicity [48], neurotoxicity [49], cardiotoxicity [50], hepatotoxicity [51], and nephrotoxicity [52]. DEHP belongs to the high molecular weight phthalate esters (HMWPE) [53]. Although phthalates are well-known as parts of plasticizers and bacterial metabolites, surprisingly, some species of the genus *Phyllanthus* of the family *Euphorbiaceae* (*Phyllanthus muellerianus*) have been reported to produce phthalates (bis (2-ethyloctyl) phthalate and bis (2-ethylicosyl) phthalate) as their secondary metabolites [54]. Additionally, Satyan et al. [55] reported that the members of the genus *Phyllanthus* have been reported to produce the phthalate compounds as their secondary metabolites.

The main fatty acids obtained from the EPME were oleic acid and palmitic acid. A similar fatty acid profile was identified in other *E. peplus* species like *E. gaillardotii* and *E. macroclada* [56]. Additionally, lucenin 2, a flavone di-C-glycosides compound with antibacterial activity [57], was identified in EPME as a minor component. Moreover, a monocyclic sesquiterpene compound known as à-Gurjunene or humulene has been found to constitute 4.36% of EPME. The potential anti-inflammatory properties of humulene have been previously reported in laboratory experiments [58].

Herein, EPME induced significant increases in the serum levels of the myocardial damage indicators such as troponin, CPK, LDH, and CKMB. Also, a significant increment in the ALP, marker enzyme for plasma membrane functionality [59], the level was noted following EPME exposure. Additionally, the EPME caused an 18.8% elevation in the levels of AST. AST is found in high concentration in the heart and consequently used as a serum marker enzyme of cardiotoxicity as its rise can be related to necrotic changes in cardiac tissue [60]. For instance, pathology comprising the cardiac muscle allows for the leakage of huge amounts of this enzyme into the blood [61]. Hence, the mild necrosis observed herein could be accountable for the elevation of the former enzyme. Similarly, crude aqueous extracts of four *Euphorbia* species including *E. balsamifera, E. heterophylla, E. hirta, E. hyssopifolia,* and *E. lateriflora* caused a significant increment in AST level following 14 days of exposure [62]. 

The notable elevation of rats serum cardiac injury markers together with the pathological perturbations in cardiac tissues in the EPME treated group could be linked to its constituents detected by GC-MS analysis. For instance, Hillman et al. [63] has reported that DEHP can be spread to heart tissue. Additionally, Posnack [64] reported that following DEHP exposure, cardiomyocytes experienced a decrease in glucose oxidation and an increase in oxygen consumption, mitochondrial mass, extracellular acidosis, peroxisome proliferator-activated receptor alpha (PPARα) expression, and myocyte fatty acid-substrate utilization. In addition, there is a strong link between both oxidative stress and inflammation and cardiovascular disease [65,66,67]. Therefore, in the existing study, the proinflammatory cytokine TNFα immunohistochemical localization was investigated in the cardiac tissue. The EPME treated group showed moderate expression of TNF-α in the cardiac myocyte indicating the role of inflammation in the cardiotoxic events. In the same line, Gourlay et al. [68] reported that DEHP initiated an inflammatory response in both human and rat. Regarding oxidative stress as the underlying mechanism, Moustafa et al., [21] verified the EPME induced oxidative stress in rats represented by the significant decline of the several antioxidant enzymes following subchronic exposure to EPME.

In the current study, EPME exposure resulted in high levels of kidney damage byproducts. This increase may reflect renal function impairment due to EPME toxicities. This was supported by the occurrence of renal lesions including congestion of renal blood vessels, hyaline casts deposition in renal tubules, and moderate perivascular fibrosis. Similarly, acute exposure to crude ethyl alcohol extract of *E. heliscopia* induced a substantial increase in creatinine, urea, and uric acid levels together with hyaline deposits in some renal glomeruli [12]. The remarkable rise of rats serum kidney injury markers together with the pathological lesions in kidney tissues in the EPME treated group could be linked to its constituents detected by GC-MS analysis. For instance, studies have revealed that male mice exposed to 1500 ppm DEHP (37.07 peak area) had chronic advanced nephropathy [52]. Peroxisome proliferator-activated receptor alpha (PPAR-alpha) was shown to mediate the subchronic toxicity of DEHP in rodents kidneys [69].

In many renal and cardiac disorders, apoptosis is frequently involved as an underlying mechanism [70,71]. Hence, in the current study, p53 immunohistochemical localization was investigated in renal and cardiac tissues due to its major role in apoptosis cascade reaction and progression in cells like kidney and heart tissue [72,73]. The intensity of the DAB staining for the anti-p53 was significantly increased in the heart and kidney of the EPME treated group suggesting the implication of p53-dependent apoptosis in the cytotoxic events due to EPME. But, the DAB staining for the renal anti-Bax was faintly expressed. To elucidate this finding, it should be mentioned that there are several known p53 target genes including Mdm-2 [74], Bax [75], Waf-1 [76], Gadd45 [77], and PCNA [78]. However, p53 activation could preferentially induce endogenous expression of certain, but not all, target genes. For instance, Sun et al. [79] reported that 1,10-phenanthroline, a heterocyclic organic compound, differentially induces endogenous expression of several known p53 target genes such as Waf-1 and Mdm-2, but not Bax, Gadd45, and PCNA. Hence, a similar pathway could have occurred here with EPME. 

On the other hand, one of the essential mechanisms lying behind the immortality of cancer cells is the resistance to apoptosis. In this regard, some *Euphorbia* species have been suggested as apoptosis-inducing anticancer agents like *E. hirta* [26], *E. lunulata* [27], and *E. esula* [80]. The most insightful correlation between p53-mediated transactivation and apoptosis is through its ability to induce the transcription of the pro-apoptotic members of the Bcl-2 family and the subsequent caspase activation [81]. MDM2 suppresses p53 activity either through inhibition of its transcriptional activity [82], ubiquitin ligase activity of MDM2 that promotes p53 proteasomal hydrolysis [83,84] or by interacting with the P53 and expulsion from the nucleus [85]. So with the overexpression of MDM2 in cancer cells, p53 is inhibited and does not induce the expression of genes responsible for apoptosis and cell cycle arrest. Thus, finding new inhibitors of the binding between MDM2 and p53 is highly needed because of its vital role in the induction of apoptosis and cancer cell death [86]. The determination of the X-ray structure of the MDM2 bound to a wild-type p53-derived peptide was reported and exhibited three discrete sub-pockets in the binding site [87].

About seventeen amino acids residues including Leu54, Leu57, Ile61, Met62, Tyr67, Gln72, Val75, Phe86, Phe91, Val93, His96, Ile99, Tyr100, Met 50, His 73, Gly 58, and Ile101 constitutes the deep binding cavities where the p53-MDM2 inhibitors could bind. Of these amino acids, Val 93, Leu54, Gly58, Ile61, Met62, Tyr67, His96, Ile99, and Tyr100 have been identified as the most vital based on the earlier experimental studies [87,88,89]. The valine 93 side-chain lies in the upper middle part of p53 binding cavity forming a solid point for the hydrophobic aromatic group to make a van der Waals as an exit vector for substituents to bind with the three sub-pockets (Phe19, Leu26, and Trp23) of the binding cavity [90]. This concept has led to the identification of the important highly potent MDM2 ligands including the isoquinoline clinical candidate; NVP-CGM097, and the bicyclic pyrazole-based highly potent ligand; S-enantiomer of 2,4-dimethoxy pyrimidine compound (15a) [91,92]. The results of the preclinical tumor models of compound 15a revealed that one hundred and three cell lines were categorized as sensitive. In addition, it exhibited excellent in vivo efficacy in SJSA1 tumor model after oral administration [93].

Co-crystallization of 15a the dihydropyrrolo-imidazole core of the compound creates van der Waals contacts with the central MDM2 residue V93 and the pyrrolo-oxygen receives an H-bond from the side-chain of H96. The chloro-phenyl moieties inhabit the Trp 23 and Leu 19 sub-pockets. In the Leu26 sub-pocket, it forms π–π bond with H96. The 2-methyl in the Trp23 sub-pocket contributes a pseudo-H-bond to Leu54. In addition, the compound 15a contributed in advantageous van der Waals interactions with the side-chains of residues M62, Y67, and Q72 of the MDM2 protein [91,92]. Our docking simulations showed that seven compounds displayed a relatively more stable binding affinity to MDM2 in terms of binding energy as compared to the known inhibitor 15a ligand. A further analysis was performed for assessing the orientation of *Euphorbia* compounds when interacting with the active site of MDM2. This analysis illustrates an essential part of evaluating the potential of these compounds for inhibiting MDM2. 

It is apparent that the highest contact frequencies were for the residues HIS 96 and MET 62. The DEPH compound has got a nice filling to the p53 binding pocket in addition to contact with the same binding residues, namely, MET 62 with one hydrogen bond and HIS 96 with 2 H-π interactions, of the 15a inhibitor ligand. Lucenin 2 compound showed an interesting binding pattern inside the MDM2 pocket, having good shape complementarily, and very high affinity with the binding cavity. It follows the valine principle as it forms three hydrophobic hydrogen bonds; two as hydrogen acceptors with the side chain of HIS 96 and GLN 24 in the LEU 26 sub-pockets, and one as a hydrogen donor with the VAL 93 amino acid. Glyceryl linolenate exhibited also a good fitting to the active site and is accepting an H-bond from the side chain of HIS 96 while donating another H-bond with ILE 19. The latter three compounds have the ideal characteristics of p53–MDM2 inhibitors where they possess large and rigid structures, even though they have hydrogen bond donors and acceptors, and lastly, these two molecules tend to be greatly hydrophobic and often contain numerous aromatic groups [94]. Herein, the docking study predicted that the EPME compounds could induce abrogation of p53–MDM2 binding and activation of p53 mediated apoptosis cascade. In addition, the strong p53 immunolabelling in cardiac and renal tissues of EPME treated rats strongly validates this hypothesis. 

## 4. Conclusions

In the light of the outcomes of the in vivo studies, it could be concluded, for the first time, that prolonged exposure of rat to EPME (500 mg/kg b. wt. orally two times per week for 65 days) could alter both cardiac and renal function significantly. Consequently, constant exposure of livestock animals to these plants may lead to health disturbances and negative consequences for livestock production and management. Also, the consumption of EPME in traditional medicines should be at appropriate doses. Another important perspective is that the in silico study using MOE predicted that EPME inhibits MDM2-mediated degradation of p53 suggesting possible anticancer potentials. Moreover, the immunohistochemical investigation of p53 in renal and cardiac tissues strongly validates the in silico prediction. Hence, more focus should be spotted on the efficacy of *E. peplus* as an apoptosis-inducing anticancer agent.

## 5. Materials and Methods 

### 5.1. Kits and Chemicals

Commercial enzyme-linked immunosorbent assays (ELISA) kits for troponin and creatine phosphokinase (CPK) were obtained from MyBioSource (San Diego, California, USA: MBS262135 and MBS721645 kits, respectively). Creatine kinase–myocardium bound (CK-MB) ELIZA kits were obtained from Kamiya Biomedical Company (12779 Gateway Dr, Tukwila, WA 98168, USA: KT-12247 kit). The lactate dehydrogenase (LDH) diagnostic kit was obtained from Spinreact Co. (Santa Coloma, Spain). Commercial colorimetric bioassay kits (BioMérieux, Marcy l’etoile, France) were purchased to determine serum aspartate transaminase (AST), alkaline phosphatase (ALP), urea, uric acid, and creatinine levels. Dimethyl sulfoxide (DMSO) was obtained from Techno pharmchem (Bahadurgarh Haryana, India). All other reagents/chemicals used were obtained from Sigma Co. (St. Louis, MO) and were of analytical grade.

### 5.2. Plant Material

Freshly collected *Euphorbia peplus* plant has been collected from their natural environments, Abo-Hammad, Sharkia, Egypt, certified and authenticated by Dr. Samir Salem Teleb, a Botanist of Taxonomy and Flora of Egypt, at the Department of plant, Faculty of Science, Zagazig University, Zagazig, Egypt. A voucher specimen (EP-2017) was deposited in the herbarium of the same department. The whole plant was dried at 40 °C, milled fine, and stored in a firmly closed bottle till extraction.

### 5.3. Preparation of Euphorbia Peplus Methanolic Extracts (EPME)

The dried powder plant material was macerated in a methanol (1:10 *w/v*) for 72 h at room temperature. Then, the macerate was filtered and the resultant filtrate was concentrated under reduced pressure and vacuum [95]. The yield of methanolic extract was 5% *w/w*. Freeze-dried extracts were collected following evaporation of the solvent in small glass bottles and kept at −20 °C for further experimentation and analysis. 

### 5.4. Gas Chromatography/Mass Spectrometry Analysis (GC–MS) of EPME

The GC-MS analysis was carried out at the Regional Center for Mycology and Biotechnology, Al-Azhar University Campus, Nasr city, Cairo, Egypt.The frozen dried extract sample was dissolved in analytical grade ethanol, filtered through a 0.2 µm membrane to be GC–MS analyzed using Trace GC1310-ISQ mass spectrometer (Thermo Scientific, Austin, TX, USA) with a direct capillary column TG–5MS (30 m × 0.25 mm × 0.25 µm film thickness). The column oven temperature was initially held at 50 °C and then increased by 5°C/min to 230 °C hold for 2 min. increased to the final temperature 290 °C by 30 °C/min and hold for 2 min. The injector and MS transfer line temperatures were kept at 250 °C, 260 °C respectively. Helium was used as a carrier gas at a constant flow rate of 1 mL/min. The solvent delay was 3 min and diluted samples of 1 µL were injected automatically using AI/AS 1310 Series Autosampler with glass screw thread vial coupled with GC in the split mode. EI mass spectra were collected at 70 eV ionization voltages over the range of *m/z* 40–1000 in full scan mode. The ion source temperature was set at 200 °C. The components were identified by comparison of their retention times and the mass to charge ratio (*m/z*) of the identified peaks with those of WILEY 09 and NIST 11 mass spectral databases. The full scan range was set at *m/z* 100–1000. In this experiment, several precautions have been taken into consideration to assure that resultant components in the GC-MS analysis belong to the extract, not the column or solvent used, including the use of analytical grade solvent of high purity together with using of a blank sample without the plant materials.

### 5.5. Animal Grouping and Experimental Design

Adult albino Wistar rats (male, 150–200 g) were purchased from the Laboratory Animal Farm. All rats were housed in stainless steel cages at a controlled temperature (21–24 °C) with a relative humidity of 50–60% and a 12-hr light-dark cycle. Throughout the study, all rat had ad libitum access to standard rodent chow and filtered water. Rats were acclimatized 2 wk prior to use in the experiment. All efforts were made to minimize the number of animals used and their suffering. At the time of the trial, rats were weighed and randomly allocated into three groups each containing 10 rats. Group I (C) Control rats received distilled water. Group II (DMSO) rats received 1 ml dimethyl sulfoxide (DMSO) 2%. Group III (EPME) rats received 500 mg EPME/kg b. wt. dissolved in DMSO 2% [21]. All treatments were two times per week for 65 days. Rats were carefully observed throughout the trial for signs of toxicity, morbidity, and mortality. The Ethics of Animal Use in Research Committee permitted all protocols involving animals here. All experimental procedures were conducted based on the NIH general guidelines for the Care and Use of Laboratory Animals in scientific investigations. The Institutional Animal Care and Use Committee of Zagazig University approved the present protocol with the reference number ZU-IACUC/2/F/133/2019. The experimenters were blinded to the treatments given to the animals and to the biochemical and histological analyses and the data analyses.

### 5.6. Sampling 

At the end of the dosing, all rats of different groups were euthanized by cervical dislocation. Blood samples were obtained from the medial canthus of the eye into a glass tube and left for 20 min to coagulate at room temperature; after centrifugation for 10 min at 3000 rpm, the resultant serum was isolated and kept at −20 °C until used in the biochemical assays defined below. At necropsy, the kidney and heart were removed and washed in physiological saline. Small portions of the kidney and heart were collected and fixed in 10% buffered neutral formalin solution for histopathological and immunohistochemical examinations. 

### 5.7. Serum Biochemical Analysis

Troponin, CPK, and CK-MB concentrations in the serum were estimated using ELIZA kits following the manufacturer’s guidelines. Serum AST, ALP, LDH, urea, creatinine, and uric acid, levels were measured by a colorimetric assay following the protocols of Murray [96], Wenger et al. [97], Pesce [98], Kaplan [99], Fossati et al. [100], and Barham and Trinder [101], respectively.

### 5.8. Histopathological and Immunohistochemical Investigation

The fixed samples of kidneys and heart were dehydrated in ascending grades of alcohol, cleared in xylene and implanted in paraffin. The samples were cast, then divided into 5 µm slices and retained onto glass slides. The slides were stained by general (H&E) and specific stain (Masson trichrome stain) and examined microscopically [102].

Heart and kidney tissue sections were de-paraffinized, treated with 3% H_2_O_2_ for 10 min, heated in 10 mM citrate buffer for 30 min at 121 °C, and then blocked for 20 min in 5% normal serum. Kidney samples were incubated with a rabbit polyclonal anti‑Bax primary antibody (1:100; sc-526; Santa Cruz Biotechnology, Inc., Dallas, TX, USA) [103] and heart samples were incubated with tumor necrosis alpha (TNFα) Antibody (1:100; sc-52746; Santa Cruz Biotechnology, Inc., Dallas, TX, USA) [104] in phosphate-buffered saline (PBS) overnight at 4 °C. In addition, the heart and kidney sections were incubated with primary antibodies specific for p53 diluted in PBS overnight at 4 °C (mouse monoclonal antibody; Clone: DO-7; Code No. M 7001; diluted 1:25; Dako, CA, USA) [105]. After three washes with PBS, the sections were incubated with a goat anti-rabbit IgG biotin-conjugated secondary antibody (1:2,000; sc‑2040; Santa Cruz Biotechnology, Inc.) at 32 °C for 20 min. After further incubation with horseradish peroxidase-labeled streptavidin, antibody binding was visualized by diaminobenzidine, and the sections were counterstained with hematoxylin [106].

### 5.9. Ligand Preparation for Drug-Likeness and Docking

**Figure 12:**
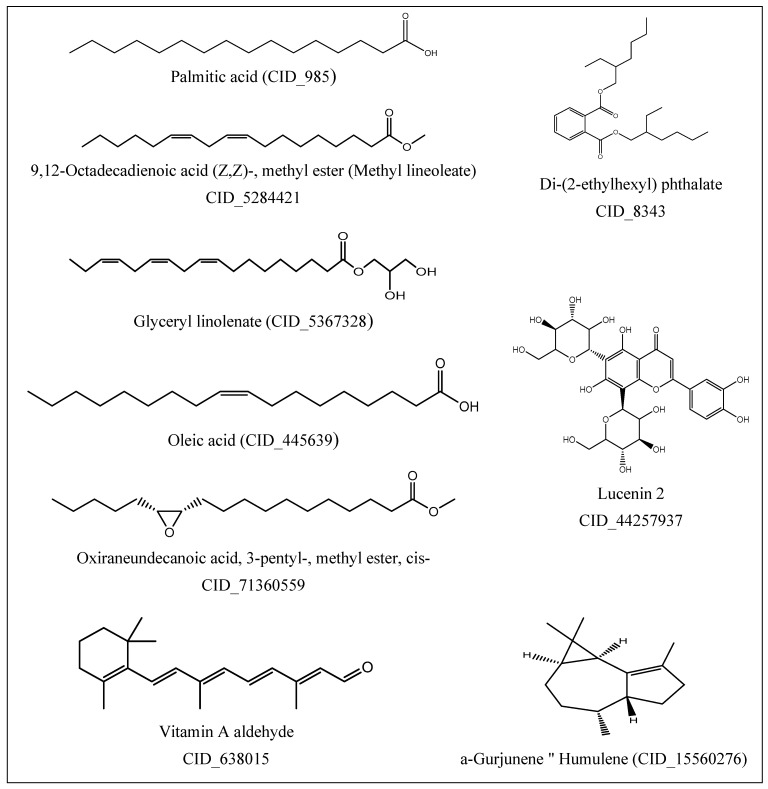
Chemical 2D structures of *Euphorbia peplus* compounds used for computational studies.

The structures of Di-(2-ethylhexyl) phthalate, oleic acid, vitamin A aldehyde, 9,12-octadecadienoic acid (Z,Z)-, methyl ester (methyl lineoleate), α-Gurjunene "humulene’’, glyceryl linolenate, lucenin 2, and oxiraneundecanoic acid, 3- pentyl-, methyl ester compounds isolated from *E. peplus* were downloaded from PubChem database. The structures of the compounds prepared and used for this study are presented in Figure 12.

The downloaded structures were then converted to three dimensional (3D) structures and the energy of them was minimized through the MM2 Force field in ChemBio3D^®^ Ultra 13.0 (Cambridge soft, USA) software. The main goal of 3D structure preparation was to correct and fix the structural data to be ready for docking.

### 5.10. MDM2 Protein Preparation for Docking

To prepare MDM2 for docking, the (3D) MDM2 structure (PDB: 6GGN) was downloaded from the protein data bank (http://www.rcsb.org) and then loaded to MOE. The preparation of the target protein involved the following processes; removal of all water molecules, the addition of hydrogen atoms, completion of residues with missing atoms, selection of appropriate alternate locations and calculation of partial charges. In MOE, these structural issues were automatically corrected using the structure preparation application.

### 5.11. Docking of Isolated Euphorbia Peplus Compounds into MDM2 Protein

To identify the molecular binding interactions of the compounds within the target protein, all the *Euphorbia* optimized compounds were docked against the 3D structure of MDM2 (PDB: 6GGN). The docking procedure was performed in MOE by default parameters. The binding site was identified by specifying the atoms of a native ligand (compound 15a) presented in the MDM2 pocket while the native ligand atoms were ignored by the software during the docking procedure.

For each molecular species, a number of placements called poses were generated and scored. The scores were then calculated as the free energy of binding (ΔGb) and the final ten highest- scoring poses for each molecule along with their scores and binding energies (ΔGb) were collated into a database. The database file generated from the docking procedure was further analyzed, with the binding interactions of the highest ten conformations for each docked molecule in the active site visualized and studied with the help of MOE visualization window. Yellow stick rendering was added to the native ligand (compound 15a) obtained from the PDB files, while *Euphorbia* compounds were marked in Red stick style for better contrast and to enable the study of the interactions of these docked compounds within the binding active site. Among the visualization of the conformation generated from the docking for each molecule, the conformation with the best binding interactions with the lowest binding energy (ΔGb) was selected for further analysis. Before the molecular docking of the *Euphorbia* compounds with MOE, docking was performed between the binding pocket of MDM2 and its native ligand (compound 15a) to validate the docking protocol by calculating the RMSD [38,107,108]. If the RMSD of the best-docked conformation of the native ligand is 2.0 Å or less from the experimental one (native ligand), the used scoring function protocol is successful [108]. The binding energy (ΔGb) and the binding mode for the native ligand were also calculated and analyzed.

### 5.12. Statistical Analysis

Data were expressed as means ± SE. Assessments of the data were done using a one-way analysis of variance (ANOVA) followed by Duncan’s multiple range test**.** A *P*-value < 0.05 was accepted as significant. All data were tested for normality via a Shapiro-Wilk W test and results reported to *P* < 5%, level of significance.

## Figures and Tables

**Table 1:** Molecular weight, chemical formula, retention time (RT) and peak area (%) of the different compounds found in *Euphorbia peplus* methanolic extract analyzed by gas chromatography/mass spectrometry analysis.

Compound	MW ^1^	Formula	RT (min)	Peak Area%
Di-(2-ethylhexyl) phthalate	390	C_24_H_38_O_4_	51.23	37.07
9-Octadecenoic acid “oleic acid”	282	C_18_H_34_O_2_	45.50	28.82
Hexadecanoic acid “palmitic acid”	256	C_16_H_32_O_2_	42.07	15.74
Lucenin 2	610	C_27_H_30_O_16_	48.77	4.54
à-Gurjunene “humulene”	204	C_15_H_24_	29.89	4.36
9,12,15-Octadecatrienoic acid, 2,3-dihydroxypropyl ester “glyceryl linolenate”	352	C_21_H_36_O_4_	43.84	3.64
Oxiraneundecanoic acid, 3-pentyl-, methyl ester, cis-(CAS)	312	C_19_H_36_O_3_	45.72	2.23
9,12-Octadecadienoic acid (Z,Z)-, methyl ester	294	C_19_H_34_O_2_	43.69	2.24
Vitamin A aldehyde	284	C_20_H_28_O	35.44	1.36

^1^ MW, molecular weight.

**Table 2:** Effects of *Euphorbia peplus* methanolic extract (500 mg/kg b. wt. orally two times per week for 65 days) on serum cardiac injury markers in Sprague–Dawley rats.

Parameters	Control	DMSO Treated	EPME Treated	*P*-Value
Troponin I (ug/L)	0.01 ^b^ ± 0.001	0.02 ^b^ ± 0.005	0.07 ^a^ ± 0.016	<0.001
CPK (U/L)	314.0 ^b^ ± 2.5	350.0 ^b^ ± 53.9	652.0 ^a^ ± 9.7	<0.001
CK-MB (ng/mL)	6.0 ^b^ ± 0.1	8.7 ^b^ ± 0.1	18.7 ^a^ ± 1.7	<0.001
LDH (U/L)	1698.3 ^b^ ± 11.9	2224.0 ^b^ ± 279.1	3853.7^a^ ± 144.7	<0.001
AST (U/L)	92.5 ^b^ ± 1.0	89.6 ^b^ ± 2.1	109.9 ^a^ ± 3.9	0.001
ALP (U/L)	42.2 ^b^ ± 1.2	54.9 ^b^ ± 1.7	81.7 ^a^ ± 7.4	<0.001

Means within the same row carrying different superscripts (a,b) are significantly different. (a) denotes the highest mean. Values shown are means ± SE. n = 5/group. CPK, creatine phosphokinase; CK-MB, creatine kinase–myocardium bound; LDH, lactate dehydrogenase; AST, aspartate transaminase; ALP, alkaline phosphatase.

**Table 3:** Interaction of MDM2 with compounds from *Euphorbia* and the native ligand (Compound 15a).

N	Compound		ΔGb	RMSD	No. of Binding Interactions	H-Bonds		π-Interactions	
	Name	PubChem CID				Type	Amino Acid	Type	Amino Acid
1	Ligand	-	−8.172	0.49	3	H-acceptor	HIS 96	pi-pi	HIS 96
						H-donor	MET 62		
2	Palmitic acid	985	−7.916	-	1	H-donor	HIS 73	-	-
3	Di-(2-ethylhexyl) phthalate	8343	−12.448	-	3	H-donor	MET 62	H-pi	HIS 96
								H-pi	HIS 96
4	Oleic Acid	445639	−12.244	-	1	H-acceptor	HIS 96	-	-
5	Vitamin A aldehyde	638015	−9.412	-	1	-	-	H-pi	TYR 67
6	9,12-Octadecadienoic acid (Z,Z)-, methyl ester (Methyl lineoleate)	5284421	−10.9625	-	1	H-acceptor	HIS 96	-	-
7	Glyceryl linolenate	5367328	−14.496	-	2	H-donor	ILE 19	-	-
						H-acceptor	HIS 96	-	-
8	α-Gurjunene “Humulene”	15560276	−6.371	-	1	-	-	H-pi	HIS 96
9	Lucenin 2	44257937	−20.733	-	3	H-donor	VAL 93	-	-
						H-acceptor	GLN 24	-	-
						H-acceptor	HIS 96	-	-
10	Oxiraneundecanoic acid, 3-pentyl-, methyl ester, cis-(CAS)	71360559	−12.626	-	1	H-acceptor	GLN 24	-	-
